# Metabolic dysfunction–associated fatty liver disease (MAFLD): an update of the recent advances in pharmacological treatment

**DOI:** 10.1007/s13105-023-00954-4

**Published:** 2023-03-28

**Authors:** Paloma Sangro, Manuel de la Torre Aláez, Bruno Sangro, Delia D’Avola

**Affiliations:** 1 Liver Unit Clínica, Universidad de Navarra, Madrid, Spain; 2 Centro de Investigación Biomédica en Red de Enfermedades Hepáticas y Digestivas, Pamplona, Spain

**Keywords:** MAFLD, NAFLD, NASH, Drug therapies, Clinical trials

## Abstract

Metabolic dysfunction–associated fatty liver disease (MAFLD) is nowadays considered the liver manifestation of metabolic syndrome. Its prevalence is increasing worldwide in parallel to the epidemic of diabetes and obesity. MAFLD includes a wide spectrum of liver injury including simple steatosis and non-alcoholic steatohepatitis (NASH) that may lead to serious complications such as liver cirrhosis and liver cancer. The complexity of its pathophysiology and the intricate mechanisms underlying disease progression explains the huge variety of molecules targeting diverse biological mechanisms that have been tested in preclinical and clinical settings in the last two decades. Thanks to the large number of clinical trials of the last few years, most of them still ongoing, the pharmacotherapy scenario of MAFLD is rapidly evolving. The three major components of MAFLD, steatosis, inflammation, and fibrosis seem to be safely targeted with different agents at least in a large proportion of patients. Likely, in the next few years more than one drug will be approved for the treatment of MAFLD at different disease stages. The aim of this review is to synthesize the characteristics and the results of the most advanced clinical trials for the treatment of NASH to evaluate the recent advances of pharmacotherapy in this disease.

## Introduction

### Definition and epidemiology

Metabolic dysfunction–associated fatty liver disease (MAFLD) has become one of the most relevant forms of chronic liver disease worldwide due to the progressively increased in obesity rates over the past 30–40 years [1]. Obesity is defined as a body mass index ≥ 30 kg/m^2^ and the World Health Organization estimates that over 13% of the world population (> 600 million people) suffer from it, with more prevalent rates among children and adolescents [2]. The metabolic syndrome includes three out of the following conditions: abdominal obesity, hypertriglyceridemia, low HDL cholesterol, arterial hypertension, and/or hyperglycemia. MAFLD is not only associated to those comorbidities but also to insulin resistance. Indeed, the nomenclature of non-alcoholic fatty liver disease (NAFLD) has been updated to MAFLD. This term describes better the liver disease associated with known metabolic dysfunction. In fact, the new definition of MAFLD refers to hepatic steatosis in addition to one of the following three criteria: overweight/obesity, presence of type 2 diabetes mellitus, or evidence of metabolic dysregulation. The exclusion of other liver diseases including alcoholic, autoimmune, or viral hepatitis is not a prerequisite for the diagnosis of MAFLD [3].

Within the general population, the overall global prevalence of NAFLD (defined using imaging criteria) is estimated to be 25% [4]. However, important variability was observed according to geographic regions, being up to 31.8% in the Middle East, 30.4% in South America, and 13.5% in Africa [4]. NAFLD incidence has rarely been measured.

Given the common risk factors between NAFLD and cardiovascular risk factors, cardiac-related death is one of the leading causes of death for NAFLD patients [5]. Other causes include liver-related death, malignancy, or other causes such as infections, type 2 diabetes mellitus, or pulmonary embolism.

NAFLD is defined as the presence of steatosis in > 5% of hepatocytes in the absence of other competing chronic liver diseases and without significant alcohol consumption (< 20 g/day in women and < 30 g/day in men). NAFLD includes non-alcoholic fatty liver (NAFL) and non-alcoholic steatohepatitis (NASH), the latter being more severe as it includes hepatocyte damage with the risk of subsequent development of significant fibrosis, cirrhosis, and/or hepatocellular carcinoma [6]. In this context, NASH has become one of the leading causes of cirrhosis in adults in the USA and NASH-related cirrhosis is currently the second indication of liver transplantation in the USA [7].

Whereas the definitive diagnosis of both MAFLD and NASH requires a liver biopsy, imagine techniques such as magnetic resonance imaging (MRI) (MRI proton density fat fraction [MRI-PDFF] or magnetic resonance spectroscopy), computed tomography (CT), ultrasound (US) with controlled attenuation parameter (CAP), and/or elastography may provide a valuable assessment of fat deposition and significant fibrosis [6]. Although no specific treatments other than the control of the associated metabolic disorders is available, follow-up to control comorbidities is recommended to detect disease progression.

### Genetics and pathogenesis

Most individuals with MAFLD have no symptoms, and the disease may remain silent until it has progressed to cirrhosis [8]. Recent data suggest that the transition from NAFL to NASH is quite dynamic whereas fibrosis development is significantly slower in NAFLD than in NASH, needing even up to 14 years [9]. Up to 20% of patients with NASH are considered as “rapid progressors” where, although predictors are largely unknown, genetic susceptibility might play a role [10]. The relevance of the genetic determinants is just beginning to be elucidated [11], but they may involve intrahepatic lipolysis, triglyceride export, hepatic mitochondrial oxidation, or glucokinase activity. For instance, a significant association of a variant in “patatin-like phospholipase domain-containing 3” (*PNPLA3*) gene on chromosome 22 leading to modifications in retinol metabolism [12]; or in “transmembrane 6 superfamily member 2” (*TM6SF2*) gene on chromosome 19 leading to an impaired lipid transporter, have been associated with fatty liver disease [13]. Polymorphism in “ectoenzyme nucleotide pyrophosphate phosphodiesterase 1” (*ENPP1* or *PC1*) and in “insulin receptor substrate-1” (*IRS1*) genes, which are related to insulin resistance, have also been described in NAFLD patients [14]. Even, a polymorphism in “membrane bound O-acyltransferase domain-containing 7” (*MBOAT7-TMC4*) gene, involved in oxidative stress, increases the risk of fibrosis in patients with NAFLD [15]. Genetic variants in “glucokinase regulatory protein” (*GCKR*) gene [16], “solute carrier family 2-member 1” (*SLC2A1*) gene [17], and “17-beta hydroxysteroid dehydrogenase 13” (*HSD17B13*) gene [18], have also been identified in patients with fatty liver disease.

NASH is the result of multiple intracellular signals derived either from proinflammatory molecules and contact with immune cells and/or from external stimulation through visceral adipose tissue and gut microbiome, the last two conditioned by diet. The outcome of these interactions on hepatocytes includes modified insulin signaling, lipogenesis, mitochondrial dysfunction, and activation or abnormal functioning of nuclear receptors such as bile acid receptors farnesoid X receptor (FXR) [19], liver X receptor (LXR) [20], pregnane X receptor (PXR) [21], and vitamin D receptor (VDR) [22] leading to hepatic inflammation by different downstream effects. Hepatic stellate cells are activated by these signals may trigger a fibrogenic response with extracellular matrix production [23].

Diet with excess carbohydrates and saturated fat lead to an energy metabolism imbalance with lipid deposition in skeletal muscle. Subsequent muscle insulin resistance due to increased intramyocellular lipid content impedes the storage of ingested glucose as muscle glycogen [24]. Glucose is then rerouted to the liver where insulin resistance stimulates sterol regulatory element–binding protein 1c (SREBP1c) [25] which increases the expression of hepatic enzymes that regulate de novo lipogenesis with higher VLDL production and hypertriglyceridemia. Other transcription factors such as carbohydrate-responsive element–binding protein (ChREBP) [26], peroxisome proliferator–activated receptor gamma coactivator 1-beta (PPARg coactivator 1-b) [27], and LXR [28] also foster liver lipogenesis.

Although visceral adipose tissue has been related to the pathogenesis of NASH, visceral adipose tissue may just be an additional site for lipid accumulation when subcutaneous adipose tissue capacity is surpassed [29]. Circulating triglyceride-enriched lipoproteins are cleared by peripheral lipoprotein lipase (Lpl) [30]. However, an increase in endogenous Lpl inhibitors, such as apolipoprotein C3 (ApoC3) [31], angiopoietin-like proteins 3/8 (ANGPTL3/8) complex, and ANGPTL4 [32], impedes clearance of these circulating triglyceride-enriched lipoproteins and therefore, induces an increased hepatic triglyceride uptake. Increases in endogenous Lpl inhibitors have been described in NASH patients.

All in all, NASH is a heterogeneous disease with a complex pathophysiology that makes finding a single effective treatment a major challenge.

## Drug therapy in NASH

It is well-known that the first approach to treat NAFLD patients is the nutritional intervention with a change in lifestyles. However, the complexity of NASH pathophysiology and the interplay of multiple genetic and environmental factors in disease progression explains why the pharmacotherapy of this clinical condition includes a variety of molecules with different biological targets, from drugs used to treat of the diseases that contribute to NASH development and progression (i.e., antidiabetic agents) to drugs targeting liver inflammation and fibrosis. Its long natural history and wide spectrum of severity, from mild inflammation to end-stage cirrhosis, are challenges in the evaluation of pharmacological interventions and requires specific interventions for each disease stage.

**Fig. 1:**
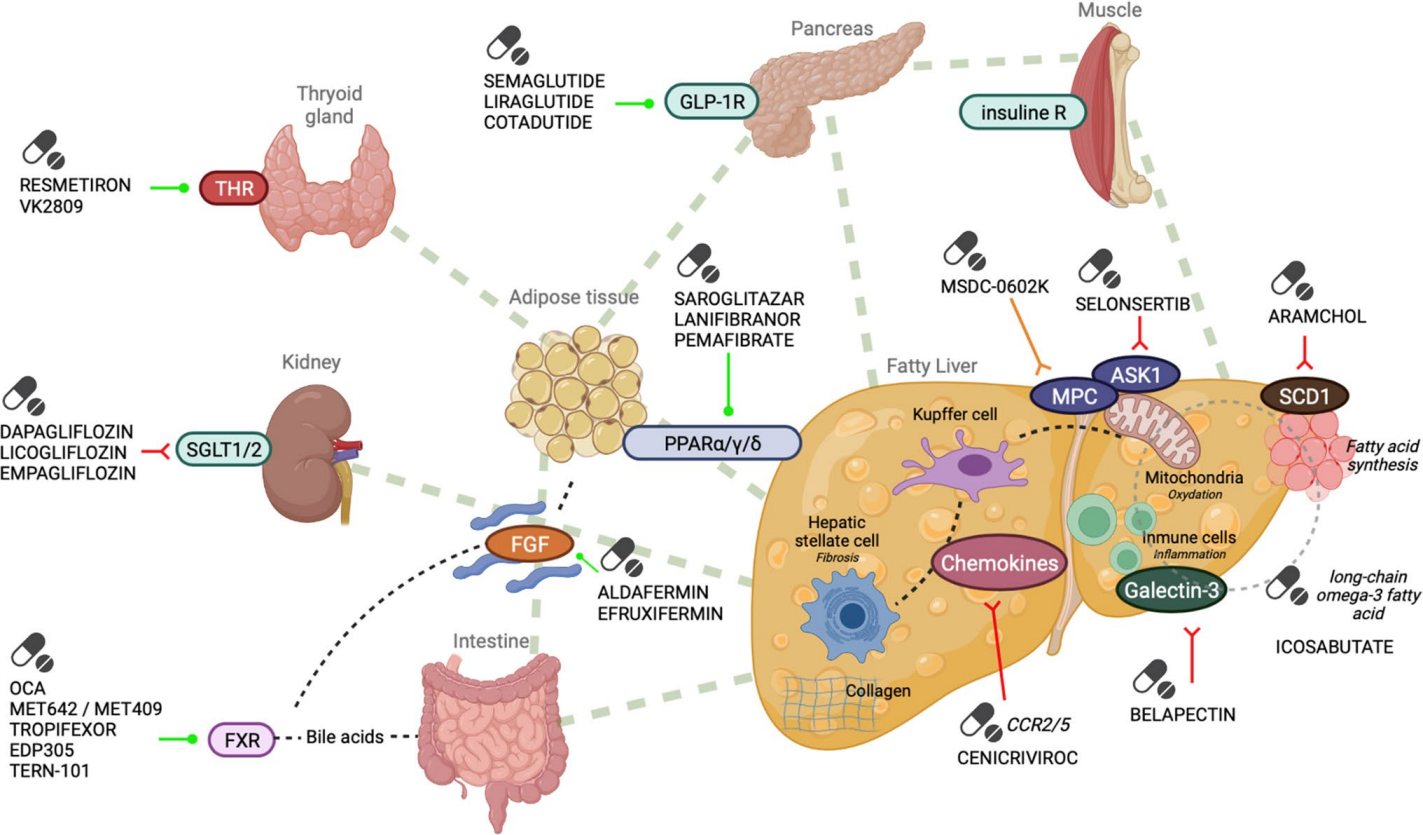
Main drugs and targets in NASH treatment

In the last 10 years, more than 20 molecules have been tested for the treatment of NASH. Most drugs were discarded after unsuccessful clinical trials, others are still in early stage of clinical development (phase 1 and 2 clinical trials) and a few have reached phase 3 trials. So far, drugs at most advanced clinical development include antidiabetic drugs, FXR agonists, PPAR agonists, and thyroid hormone receptor (THR) agonists (Fig. 1). Table 1 includes a synthesis of the clinical trials (phase II/III) in treatment of NASH.

**Table 1:** Main clinical trials in treatment of NASH/NAFLD (phase II/III)

Molecule and main trial	Mechanism of action	Primary objective	Patients recruited (*n*)	Grade of fibrosis	Main results	Most frequent adverse events
New anti diabetic agents						
Dapaglifozin *DEAN study* *(NCT03723252) phase 3*	Inhibitor of SGLT2	Improvement in scored liver histology over 12 months	100	NA	*Recruiting*	
Semaglutide *(NCT02970942) phase 2*	Agonist GLP1	NASH resolution without worsening of fibrosis	320	2- 3	NASH resolution No fibrosis improvement	Nausea, diarrhea, abdominal discomfort, reaction site injections
Semaglutide *ESSENCE study* *(NCT04822181) phase 3*	Agonist GLP1	NASH resolution without worsening of fibrosis Improvement of fibrosis without worsening NASH	1200	2–3	*Recruiting*	
Liraglutide *LEAN trial* *(NCT01237119) phase 2*	Agonist GLP1	Histological resolution of NASH	26	NA	NASH resolution with worsening of fibrosis	Nausea, diarrhea, abdominal discomfort, reaction site injections
PPAR modulators						
Pioglitazone *AIM 2 trial* *(NCT04501406) phase 2*	PPARγ agonist	Improvement in scored liver histology over 72 weeks	166	1–3	*Recruiting*	
Pioglitazone/vitamin E *PIVENS trial* *(NCT00063622) phase 3*	PPARγ agonist	Improvement in scored liver histology over 96 weeks	247	NA	Vitamin E vs. placebo: improvement Pioglitazone vs. placebo: no improvement	
Saroglitazar *EVIDENCES II study (NCT03061721) phase 3*	Dual PPARα and PPARγ agonist	Reduce ALT from baseline	104	0–3	Improvement of NASH by liver biopsy after 52 weeks of treatment Reduction of ALT from baseline	Diarrhea and cough
Lanifibranor *NATIVE study* *(NCT03008070) phase 2*	Pan-PPAR agonist	Histological resolution of NASH	247	0–3	Improvement of NASH and fibrosis by liver biopsy	Nausea, diarrhea, peripheral edema, anemia, and weight gain
Elafibranor *RESOLVE-IT study* *NCT02704403) phase 3*	Dual PPARα and PPARδ agonist	NASH resolution without worsening of fibrosis	2157	1–3	No improvement in NASH	
Pemafibrate *(NCT03350165) phase 2*	PPARα agonist	Percentage change in liver fat content measured by MRI from baseline to week 24	118		No differences in liver fat content but there was a reduction in stiffness measured by MRI	Mild-or-moderate adverse events
FXF agonist						
Cenicriviroc *AURORA trial* *(NCT03028740) phase 3*	CCR2/CCR5 agonist	Improvement in liver fibrosis by ≥ 1 stage and no worsening of steatohepatitis on liver histology	2000	2–3	Ended trial by interym analisys and lack of efficacy	Well tolerate in general way, but safety was no reported in Aurora trial
Selonsertib *Stellar 3–4, (*PMID: 32,147,362*) (fail)*	Inhibitor of ASK1				Trial fails in this primary objective	
Other metabolic treatments						
Aramchol *ARREST study* *(NCT02279524) phase 2*	Partial inhibitor of hepatic stearoyl-CoA desaturase	Hepatic fat fraction measured by MRI	247	0–3	No changes in liver fat by MRS. Improvement in liver fibrosis by ≥ 1 stage and no worsening of NASH on liver histology	Well tolerated
**PXL065** *(NCT04321343) phase 2*	Inhibitor of mitochondrial pyruvate carrier and acyl-CoA synthetase 4	Hepatic fat fraction measured by MRI	123	1–3	Improvement in liver fat content and improve in fibrosis stage	

### Antidiabetic drugs

As insulin resistance and type 2 diabetes mellitus are closely associated with NASH, the efficacy of several antidiabetic agents has been studied in NASH. Pioglitazon**e** showed histological benefits (improved NAFLD activity score, also called NAS or improvement in single histological components of NASH) in three randomized trials in diabetic and prediabetic patients with NASH. However, the effect on liver fibrosis was not significant, although worsening of liver fibrosis was not observed [33–35].

Newer antidiabetic agents including SGLT1/2 (sodium-glucose co- transporter-1/2) inhibitors and GLP-1R (glucagon-like peptide 1 receptor) agonists are being tested in NASH. Dapagliflozin and semaglutide have reached late-stage clinical development.

Dapagliflozin is an oral SGLT2 inhibitor which impedes glucose reabsorption in the proximal tubule leading to glucosuria and plasma glucose reduction. Between 2018 and 2021, many studies regarding the use of dapagliflozin in NASH patients were published. A meta-analysis of 7 trials showed that treatment with 10 mg dose dapagliflozin compared to the placebo or control group in patients with NASH (image-based diagnosis) significantly lowered ALT (weighted mean difference (WMD): − 6.62U/L; 95%CI: − 12.66, − 0.58; *p* = 0.03) and AST levels (WMD: − 4.20U/L; 95%CI: − 7.92,-0.47; *p* = 0.03). Gamma-glutamyl transferase (GGT) levels were non-significantly decreased whereas homeostatic model assessment of insulin resistance (HOMA-IR) was significantly affected (WMD: − 0.88; 95%CI: − 1.43,-0.33; *p* = 0.002). Although levels of total cholesterol increased under dapagliflozin treatment, safety profile between groups had no significant difference [36]. The DEAN study (NCT03723252) is a phase 3 trial which compares dapagliflozin vs. placebo among patients with histologically confirmed NASH with the primary endpoint of improvement in scored liver histology at 12 months. Other endpoints include resolution of NASH, changes in fibrosis score and changes in metabolic features such as body weight, hemoglobin A1 (Hb1Ac) or insulin resistance.

Other SGLT1/2 inhibitors are in earlier phases of clinical testing in NASH. The ELIVATE study (NCT04065841) is assessing if licogliflozin alone or in combination with tropifexor, an agonist of the bile acid receptor FXR, improves fibrosis and/or NAS score in patients with NASH and fibrosis stage 2 or 3. A phase 2a study to evaluate the safety and tolerability of MET409 (a synthetic FXR agonist) alone or in combination with empagliflozin in patients with type 2 diabetes mellitus and NASH has finalized recruitment and the results will be reported soon.

Semaglutide is a GLP-1R agonist which enhances insulin secretion to regulate blood glucose level. This GLP-1R agonist was first assessed in a 72-week phase 2 clinical trial [37] in patients with biopsy-confirmed NASH and liver fibrosis stages 1, 2, or 3. Patients were randomly assigned to receive semaglutide at a dose of 0.1 mg (80 patients), 0.2 mg (78 patients), or 0.4 mg (82 patients) or placebo (80 patients). Primary outcome was NASH resolution without worsening of fibrosis and only patients with stage 2 or 3 fibrosis levels were assessed in this cohort. This outcome was achieved in 40% of patients in the 0.1-mg group, 36% in the 0.2-mg group, 59% in the 0.4-mg group, and 17% in the placebo group (*p* < 0.001 for semaglutide 0.4 mg vs. placebo). However, no significant differences were observed between the different doses used and the percentage of patients with an improvement in fibrosis staging (43% with 0.4 mg vs. 33% with placebo, *p* = 0.48). Gastrointestinal and gallbladder disorders occurred in a higher percentage of patients in the semaglutide groups. Increases in amylase and lipase from baseline were also observed in the treated groups without any acute pancreatitis episode. A phase 3 clinical trial (ESSENCE, NCT04822181) is recruiting non-cirrhotic NASH patients to evaluate the potential resolution of steatohepatitis and the improvement in fibrosis with this GLP-1R agonist.

The LEAN trial [38], a phase 2 trial evaluating the effect of liraglutide (1.8 mg daily) compared to placebo in patients with biopsy-confirmed NASH, showed histological resolution of NASH (39% with liraglutide vs. 9% with placebo, *p* = 0.019). Fibrosis progression was observed in 36% of patients receiving placebo *vs* in 9% of liraglutide-treated patients (*p* = 0.04). Adverse events reported were mainly gastrointestinal disorders or administration site reactions.

Others GLP1R such as cotadutide (NCT04019561), tirzepatide (NCT04166773), or efinopegdutide (NCT04944992) have been tested or are still being tested in phase 2 trials and the results have not been published yet.

### PPAR modulators

The peroxisome proliferator-activator receptor (PPAR) family is formed by PPARα, PPARγ, and PPARδ which are mainly located in liver, brown adipose tissue, and macrophage. They activate fatty acid oxidation, lower synthesis of triglycerides, and increase insulin sensitivity. Saroglitazar is a dual PPARα and PPARγ agonist, that was first approved in India for the treatment of type 2 diabetes mellitus patients with hypertriglyceridemia [39]. Since 2020, it is widely used as treatment for NASH patients in India. Among patients with NAFLD (stages 0–3) diagnosed by imaging (ultrasound, CT scan, or MRI) or liver biopsy showing NASH or simple steatosis, and alanine aminotransferase (ALT) > 1.5 upper limit of normal recruited into a phase 3 trial (EVIDENCES II), saroglitazar reduced ALT levels after 16 weeks of therapy [40]. Patients receiving saroglitazar 4 mg reduced the most their levels of ALT (− 45.8% vs. 3.4%, *p* < 0.001). Histological improvement of NASH after 16 weeks of treatment was observed with 4 mg of saroglitazar (reduction in liver fat content − 19.7% vs. 4.1% with placebo (*p* = 0.004)). Safety and tolerability of saroglitazar were further assessed in a phase 2 study (EVIDENCES IV), and the most frequent adverse effects were diarrhea and cough [41].

After a successful phase 2 b trial, elafibranor, a dual PPAR-alpha/delta agonist, was tested in a phase 3 clinical trial in patients with non-cirrhotic NASH. In this trial, elafibranor did not meet histological endpoints (NASH resolution without worsening of fibrosis) nor the key secondary endpoint (fibrosis improvement at least one stage) [42].

Lanifibranor, the only pan-PPAR agonist, was assessed in non-cirrhotic biopsy-confirmed highly active NASH (stages 0–3) patients in a phase 2b trial (NATIVE study) were 1200 mg showed better results than 800 mg dose or placebo in histological resolution of NASH (49% and 39%, respectively, vs. 22%), histological improvement of fibrosis (48% and 34%, respectively, vs. 29%), or both (35% and 25%, respectively, vs. 9%) were observed [43]. Nausea, diarrhea, peripheral edema, anemia, and weight gain occurred more frequently with lanifibranor than with placebo.

The PPARα agonist pemafibrate has been tested in NASH patients (MRI diagnosis with ALT elevation) [44]. The primary endpoint of percentage change in liver fat content measured by MRI at week 24 was not met (− 5.3% vs − 4.2%; treatment difference − 1.0%, *p* = 0.85). However, liver stiffness measured by MRI decreased at week 48 (treatment difference − 5.7%, *p* = 0.036), and was maintained at week 72 (treatment difference − 6.2%, *p* = 0.024).

### FXR agonists

FXR modulation may have multiple implications in the treatment of NASH since, besides bile acid (BA) synthesis, this receptor is involved in glucose and lipid metabolism, and in the regulation of inflammation [45]. FXR activation downregulates bile acid synthesis through the upregulation of fibroblast growth factor (FGF)-19 expression and by reducing the expression of CYP7A1, the rate-limiting enzyme of the BA synthesis. This results in a protective effect against the toxic accumulation of BAs through increased conjugation in the liver and secretion into the bile canaliculi. FXR activation improves glucose tolerance by reducing hepatic gluconeogenesis and increasing glycogen synthesis [46]. Additionally, FXR activation reduces fat accumulation in the liver by targeting SHP expression and CYP7A1 activity [47]. Obeticholic acid (OCA) is a semi-synthetic bile acid analog of chenodesoxicholic acid with a potent agonist activity on the FXR in the liver and in the intestine. OCA, as the other members of FXR agonists family, may exert multiple therapeutic effects on NASH by improving hepatic lipid and glucose metabolism and through its anti-inflammatory and anti-fibrotic activity. OCA lowers plasma triglycerides by downregulating the expression of SREBP1c expression (48) and increases hepatic fatty acid oxidation through upregulation of pyruvate dehydrogenase kinase 4 (PDK4) and modulating glucose-dependent lipogenic genes [49]. It has been shown that OCA reduces insulin resistance and improves glucose homeostasis in diabetic patients with NASH [50]. OCA may also contribute to reduce portal pressure by increasing nitric oxide synthases (iNOS) and decreasing inflammation mediators.

After a phase 2b clinical trial in patients with biopsy-proven NASH without cirrhosis (FLINT trial) in which OCA displayed a beneficial impact on fibrosis without resolution of advanced fibrosis and at least of 2 points improvement in NASH compared to placebo [51], this drug is now being tested in a large phase 3 randomized, placebo-controlled trial (REGENERATE trial, NCT2548351) to evaluate the long-term effects on NASH and fibrosis in patients with stage 1–3 fibrosis. The results of an *interim* analysis showed a significant improvement of fibrosis in approximately 20% of patients treated with OCA vs. 12% of placebo [52]. The NASH resolution endpoint was not met in this preliminary analysis. The most significant side effect was a dose-dependent pruritus, that in some cases (< 10%) required treatment discontinuation. Increased cholesterol levels were frequently observed, and statins were newly prescribed in almost 50% of patients receiving OCA [52].

At the same time, the antifibrogenic effect of OCA is being tested in a phase 3 trial including patients with compensated cirrhosis due to NASH (REVERSE trial, NCT03439254).

MET642 and MET 409 are other structurally optimized synthetic FXR agonists. Preliminary reports suggest that these compounds produce less pruritus than OCA. MET642 is now being tested in a phase 2 clinical trial in patients with NASH (NCT0477396). After the results of a phase 1b trial in which MET409 showed its ability to reduce the liver fat content in monotherapy, this drug is now being tested in a phase 2b trial in combination with empagliflozin.

Tropifexor, another highly potent non-bile acid FXR agonist, is being tested in a phase 2 clinical trial (FLIGHT FXR, NCT02855164) in patients with stage 1–3 fibrosis due to NASH. In a preliminary analysis, treatment with tropifexor lead to a significant reduction of hepatic fat, liver transaminases, and body weight compared to placebo with a favorable safety profile. Complete results are expected to be released in the next few months [53].

Cilofexor is another nonsteroidal FXR agonist that showed efficacy in decreasing liver steatosis a phase 2 trial [54]. The preliminary results of a phase 2 trial testing cilofexor in combination with firsocostat (an acetyl-CoA carboxylase inhibitor) and semaglutide suggest a synergistic activity of this combination in improving liver steatosis and liver biochemistry [55].

EDP305 is another FXR agonist. A phase 2 randomized, double-blind, placebo-controlled, dose-ranging trial (ARGON-1) has shown that when administered to non-cirrhotic biopsy-proven NASH patients, a 12-week course of EDP305 reduced liver fat content and decreased ALT [56]. The most frequent adverse event was pruritus and changes in lipid parameters were milder than with first-in-clasee FXR agonists. ARGON-2 is a phase 2b study testing the efficacy of EDP305 in NASH patients with stage 2–3 fibrosis (NCT04378010).

Finally, TERN-101 is a nonsteroidal FXR agonist with enhanced liver distribution. In a preliminary analysis of a phase 2 trial (LIFT study) in patients with stage 1–3 liver fibrosis, a 12-week course of TERN-101 rapidly decreased ALT and GGT and seemed to have positive effect on inflammation and fibrosis measured by using a non-invasive composite marker assessed by MRI (cT1) and MRI-PDFF [57].

### FGF analogs

FGF19 and FGF21 are members of FGF superfamily that exert similar but not identical beneficial effects on lipid and glucose metabolism. Indeed, the administration of both FGF19 and FGF21 in animal models improve insulin sensitivity, improve body weight and fat mass, lipid levels, and liver steatosis. This latter effect probably depends on the inhibition of SREBP1 and reduced expression of genes involved in triglyceride synthesis [58].

In the last years, different FGF21 analogs and one FGF19 analog have been tested in phase 1 and 2 trials for the treatment of NASH.

In a phase 2b trial, the FGF19 analog aldafermin failed to improve liver fibrosis among patients with NASH-related stage 2 or 3 fibrosis. Other FGF21 analogs are pegbelfermin and efruxifermin. In a phase 2 trial in patients with stage 1–3 liver fibrosis, efruxifermin reduced liver fat content, improved liver function tests, and fibrosis and inflammation markers (including NAS score). Fibrosis stage improvement of at least 1 point was observed in almost half of the patients and NASH resolution in almost one third. Safety profile was favorable [59].

### THR beta agonists

THR-β is involved in the regulation of lipid metabolism, plays a role in insulin sensitivity, and promotes liver regeneration and reduces hepatocyte apoptosis in the liver. In a phase 2 trial in patients with NASH and fibrosis, the THR-β agonist resmetirom decreased liver fat content and improved lipid metabolism parameters and liver function tests, with a positive impact on lipid profile and fibrosis markers, with no significant effect on body weight [60]. A phase 3 study on patients with non-invasive diagnosis of NAFLD showed that 100 mg of resmetiron administered daily for 52 weeks improved liver fat content in approximately 50% of treated patients *vs* 8% of placebo-treated patients; liver fibrosis in approximately 20% of treated patients vs. 10% of placebo-treated patients (both measured by a non-invasive test). Lipid metabolism parameters, liver enzymes, and inflammatory biomarkers also improved compared to placebo [61]. There were no major safety concerns. Main adverse events were gastrointestinal (diarrhea, nausea). A phase 3 study in NASH patients with fibrosis is still ongoing and data will be available soon (MAESTRO-NASH, NCT03900429).

A liver-directed THR-β agonist, VK2809, improved liver fat content compared to placebo among patients with NAFLD treated with two different doses in a preliminary analysis of a phase 2 trial [62]. No serious adverse events were reported.

### Anti-fibrotic and anti-inflammatory agents

Currently, many agents with anti-fibrotic and anti-inflammatory effects are being tested in NASH. Most of them are still in early stage of clinical development: in phase 1 (as for example GB1211 targeting galectin 3, DFV890 targeting NLPR-3, or nimacimab targeting CB1) and phase 2 (as for example tipelukast, a leucotrien, or nitazoxanide, an antiparasitic agent). The description of all of them exceeds the objective of this review.

The available information from molecules in later stages of clinical development is summarized below.

Cenicriviroc (CVC) is an orally administered, small molecule antagonist that blocks chemokine 2 and 5 receptors, both with well-known roles in liver inflammation and fibrosis. In a phase 2b trial (CENTAUR), the primary objective of histological improvement in NASH was not met although CVC improved measurable liver fibrosis without worsening NASH [59]. A phase 3 trial (AURORA, NCT03028740) was prematurely stopped when an interim analysis casted doubts on the efficacy of the drug. Preliminary results of a phase 2b trial (TANDEM, NCT03517540) showed that the combination of tropifexor with CVC was safe and able to reduce body weight and ALT in patients with biopsy-proven NASH with fibrosis. However, the combination was not superior to either drug in monotherapy in terms of histological endpoints [63].

Belapectin is a complex carbohydrate that targets galectin-3, a B-galactoside-binding lectin that plays a role in inflammatory response and fibrosis [64]. A phase 2b clinical trial (NASH-CX, NCT02462967) showed that belapectin has not any significant effect on inflammation and fibrosis compared to placebo. However, it produced a significant decrease of the hepatic venous pressure gradient in patients with NASH-related cirrhosis without esophageal varices [65]. A phase 2b/3 trial (NAVIGATE, NCT04365868) is assessing the proportion of NASH patients with compensated cirrhosis who develop new esophageal varices after an 18-month course of belapectin compared to placebo, as well as the incidence of long-term clinically significant cirrhosis-related events.

Selonsertib is an oral inhibitor of the apoptosis signal–regulating kinase-1 (ASK1). Although well tolerated, it failed to show improvement in fibrosis without worsening of NASH in two different phase 3 trials (STELLAR-3, in patients with stage 3 fibrosis; and STELLAR-4, in patients with compensated cirrhosis) [66].

### Other metabolic treatments

Molecules involved in other metabolic pathways are at different stage of clinical development for the treatment of NASH.

Stearoyl-coenzyme A desaturase-1 (SCD-1) is considered a mediator of liver steatosis and fibrosis because of its role in fatty acid biosynthesis [67] [68]. Aramchol is an oral SCD1 modulator which showed an ability to reduce liver fat and improve NASH and fibrosis with favorable tolerance among patients with overweight or obesity and prediabetes in a phase 2b trial (ARREST) [69]. An ongoing phase 3 trial (ARMOR study, NCT04104321) in patients with advanced fibrosis and NASH aims to evaluate the efficacy of aramchol vs. placebo on NASH resolution, fibrosis improvement, and clinical outcomes related with NASH progression.

Icosabutate is a synthetic omega 3 fatty acid (a structurally engineered eicosapentaenoic acid that resist oxidation and does not accumulate in hepatocytes) that could confer beneficial effects on hepatic oxidative stress, inflammation, and fibrosis. A phase 2b study is evaluating the efficacy of different doses of icosabutate on the resolution of NASH without worsening of fibrosis. The primary endpoint is to evaluate the percentage of patients with resolution of NASH defined as disappearance of ballooning with lobular inflammation without worsening in fibrosis (ICONA study, NCT04052516).

MSDC-0602 K is a thiazolidine-dione designed to modulate the mitochondrial pyruvate carrier (MPC), a protein complex that regulates the entry of pyruvate into the mitochondria. A phase 2b placebo-controlled randomized trial (EMMINENCE) failed to show improved histological outcomes (≥ 2-point NAS improvement without worsening fibrosis, NASH resolution, and fibrosis reduction) among patients with biopsy-proven NASH and stage 1–3 fibrosis [70]. A dose-dependent improvement in the glycemic control and liver enzymes were nevertheless observed.

A novel, deuterium-stabilized R-pioglitazone, PXL065, that lacks PPAR-gamma activity but exerts its non-genomic target activities (mitochondrial pyruvate carrier and acyl-CoA synthetase 4 inhibition) has been tested in a phase 2 trial in non-cirrhotic patients with NASH (NCT04321343). According to the preliminary results reported at AASLD 2022 meeting, this drug can reduce the liver fat content in 40% of patients and improve at least 1 fibrosis stage in 30–50% of treated patients. In this trial, up to 30% of patients showed NASH resolution after 36 weeks of treatment with a good safety profile since it lacks the PPAR-gamma side effects of glitazones [71]. The phase 3 trial has not started yet.

Other molecules targeting fatty acid synthesis or de novo lipogenesis, such as DGAT2i (NCT04399538) or TVB-2640 (NCT03938246, NCT04906421) have been tested in phase 2 trial alone or in combination with other compounds. Ad interim analysis showed encouraging results for TVB2640 but the definitive results have not been reported yet.

## Conclusion

The pharmacological scenario of NASH therapy will certainly change in the next future. The results of the latest clinical trials are showing that the three major components of NASH, steatosis, inflammation, and fibrosis can be pharmacologically targeted. Although therapies on trial have acceptable tolerance and a good safety profile, a drug with potential to ameliorate all components of NASH is yet to be identified. Indeed, the results of single drug trials, most of them preliminary, showed improvement of NASH components in only a proportion of NASH patients, in general lower than 50%. Combination therapy looks a promising strategy. The rationale behind drug combination is on one hand to increase the efficacy of one single drug and on the other hand, to reduce side effects of one drug by allowing the use of a lower dose or by controlling the side effects of the first drug (72). So, the best treatment will probably be defined in the next few years not only according to different disease stages but also aiming to tailor the treatment for each patient according to the presence of comorbidities.
